# Impaired Driving Among Rural Appalachian Women in the Year Following Incarceration

**DOI:** 10.13023/jah.0604.09

**Published:** 2025-01-29

**Authors:** J. Matthew Webster, Megan F. Dickson, Shawn M. Jamison, Michele Staton

**Affiliations:** University of Kentucky; University of Kentucky; University of Kentucky; University of Kentucky

**Keywords:** Appalachia, impaired driving, reentry, women

## Abstract

**Introduction:**

Despite the known risks associated with substance use following incarceration, no studies have examined substance-impaired driving as a post-incarceration health risk behavior in rural Appalachia.

**Purpose:**

The present study examined differences by impaired driving following incarceration and identified predictors of impaired driving in sample of rural Appalachian women with a history of drug use and risky sex.

**Methods:**

Women (N=340) from three rural Appalachian jails completed a baseline interview in jail and follow-up interviews at six and 12 months post-release. Interview questions included demographic characteristics and information on substance use risk, mental health, criminal legal system involvement, and impaired driving. Data were collected from 2012 to 2019. Secondary data analysis performed in 2024 compared women who reported driving impaired during the 12-month follow-up period (n=76) to those who did not (n=257). A multivariable logistic regression was performed to identify predictors of impaired driving.

**Results:**

Lifetime arrests, substance use risk, and symptoms of major depressive disorder were associated with impaired driving. The logistic regression model indicated that participants with major depressive disorder symptoms had twice the odds of driving impaired in the year following incarceration. Implications: Almost one-fourth of women in the sample reported driving impaired during the follow-up period, suggesting that impaired driving should be examined as a post-release health risk behavior for rural Appalachian women in future research. Furthermore, study findings highlight an important opportunity for targeted prevention and intervention for women who may experience increased risk as they return to rural Appalachian communities following incarceration.

## INTRODUCTION

Community reentry following incarceration is often characterized by a return to risk behaviors that contributed to criminal legal system involvement. Prominent among these risk behaviors is substance use. Despite the known risks associated with substance use following incarceration (e.g., recidivism, overdose),^1^ there are few studies examining impaired driving (i.e., substance-impaired driving) as a health risk behavior following incarceration.

This is a notable gap in the literature given that impaired driving is a significant threat to public safety. Because previous research indicates individuals arrested for impaired driving often have prior criminal involvement,^2^ there is a need to better understand impaired driving during community reentry, particularly among understudied populations such as those reentering rural Appalachian communities.^3^ Rural communities, in general, have disproportionately higher rates of impaired driving arrests and associated risks.^4^ Few studies, however, examine impaired driving in rural Appalachia, and none examine impaired driving among women returning to rural Appalachian communities after incarceration.

Prior work indicates that rural women are particularly vulnerable following incarceration, face an array of barriers disproportionately affecting women (e.g., stigma, trauma, and childcare), and are more likely than males to engage in health risk behaviors, including both drug- and sex-related risk behaviors.^5^,^6^ Further, recent research has found a correlation between a history of health risk behaviors and impaired driving among rural Appalachian justice-involved women.^7^

As a follow-up to a previous study published in *Traffic Injury Prevention*, which examines impaired driving in the year prior to incarceration,^8^ the current study focuses on impaired driving among rural Appalachian women with substance use histories who are reentering the community post-incarceration. The following research questions were examined:

1. What factors are associated with impaired driving among rural Appalachian women in the year following incarceration?
2. What factors best predict impaired driving among rural Appalachian women in the year following incarceration?

## METHODS

### Participants and Procedure

As part of a study designed to lower HIV risk, a sample of women (N=400) from three rural Appalachian jails in Kentucky were recruited and screened and consented to participate. Eligibility was based on 1) a NIDA-modified Alcohol, Smoking and Substance Involvement Screening Test (NM-ASSIST)^9^ score of 4+ for at least one of the seven drugs measured, indicating at least moderate risk associated with their substance use; and 2) engagement in at least one sex risk behavior in the 3 months before incarceration. Full recruitment and procedural details have been described by Staton et al.^5^

Baseline interviews were conducted with women in a private room at the jail. Research staff contacted and interviewed participants again at both six and 12 months post-release. Of those who completed the baseline, 340 completed both follow-up interviews (85% follow-up rate). Participants were paid $25 for each interview and a $25 bonus for completing all three interviews. All data were collected from 2012 to 2019. Research procedures were approved by the university IRB.

### Measures

#### Demographics

Demographic information included age, race (1 = white, 0 = non-white), years of education, relationship status (1 = married/living as married; 0 = single), employment during the six months prior to incarceration (1 = employed at least part-time; 0 = unemployed), and driver’s license status (1 = had a license, 0 = no license).

#### Substance Use

Substance use risk level was measured using the NM-ASSIST for cannabis, cocaine, prescription stimulant, methamphetamine, sedative, street opioid, and prescription opioid use in the three months prior to incarceration. Scores ranged from 0 (low risk) to 39 (high risk) for each substance. Measures for each substance had acceptable reliabilities (Cronbach’s α > .81).

#### Mental Health

The Global Appraisal of Individual Needs (GAIN)^10^ was used as a baseline measure of participants’ mental health, with a focus on anxiety, depression, and post-traumatic stress disorder (PTSD) (1=reported criteria consistent with the specified mental health problem; 0=did not report criteria consistent with the problem). Measures for each disorder had acceptable reliabilities (Cronbach’s α > .87).

#### Criminal Legal System Involvement

Self-reported total numbers of arrests and incarcerations during participants’ lifetime were used as measures of criminal legal system involvement.

#### Impaired Driving

Participants were asked about substance-impaired driving at each interview. For this study, the outcome variable was whether participants reported impaired driving during either of the two follow-up interviews (1 = yes; 0 = no). Substance-impaired driving in the year prior to incarceration was used as a control (1 = yes; 0 = no).

#### Data Analysis

Secondary data analysis was performed in 2024 using data from the HIV risk reduction study. A first set of analyses addressed the first research question and included chi-square and t*-*tests to identify differences between those who reported driving impaired during the 12-month follow-up period and those who did not. To address the second research question, a simultaneous, multivariable logistic regression model examined independent correlates of impaired driving during the follow-up period. Multicollinearity among the included variables was assessed using variance inflation factors with none greater than 1.3. Seven participants were excluded from the final sample (N=333) due to missing data.

## RESULTS

As reported during the baseline interview, participants were predominantly white (98.8%), unmarried (64.0%), and unemployed prior to incarceration (76.9%; see Table 1). The average age of participants was 32.4 (SD = 8.0; Mdn=31.0) with an average education less than high school (M *=* 11.2 years, SD=2.3; Mdn = 12.0). Despite only 35.4% reporting a valid driver’s license prior to incarceration, 66.4% indicated they had driven under the influence of a substance in the year prior to incarceration. Additionally, 22.8% reported driving impaired during the 12-month follow-up period.

Compared to their counterparts, women who reported driving impaired during follow-up indicated greater substance use risk for several substances at baseline, with significantly higher NM-ASSIST scores for cocaine (*t*(112.5) = −2.48, *p* = .015), methamphetamine (*t*(331)= −2.79, *p* = .006), and sedatives (*t*(331)= −2.37, *p* = .018). Impaired drivers were also significantly more likely to report symptoms consistent with depression (χ^2^(1, N=333)= 7.84, *p* = .005) and reported a greater number of lifetime arrests (*t*(331)= −2.35, *p* = .019). There were no demographic differences.

The multivariable logistic regression model (Table 2), including variables significant at the bivariate level, was then used to identify predictors of impaired driving. The NM-ASSIST score for methamphetamine was positively associated with the likelihood of driving substance-impaired during the follow-up period, although the confidence intervals suggest only a marginal relationship. Additionally, individuals reporting symptoms consistent with depression were twice as likely to drive substance impaired.

## IMPLICATIONS

The present study examines factors associated with impaired driving after jail incarceration among rural Appalachian women with substance use and risky sex histories. Although demographic characteristics appear unrelated to impaired driving post-incarceration, the number of lifetime arrests is associated with a greater likelihood of driving impaired, which is consistent with previous studies.^2^ Women with greater cocaine, methamphetamine, and sedative risk scores are also more likely to drive impaired. Given the number of problems ascribed to the opioid epidemic in rural Appalachia,^3^ it is interesting to note that opioid risk scores are unrelated to impaired driving. While all three mental health disorders are more prevalent for impaired drivers, only major depressive disorder symptoms significantly differ between groups. Other studies also have found a link between depression and impaired driving.^11^,^12^

Results from the logistic regression indicate that major depressive disorder symptoms are the only significant predictor of impaired driving among the examined variables. Individuals with depression often self-medicate with substances, which likely contributes to their impaired driving.^12^ Future research should more closely examine how depression may influence rural Appalachian women’s likelihood of impaired driving during community re-entry, including how interventions can be better tailored to address their needs during this time. For example, interventions using technology (e.g., telehealth, mHealth) designed to increase women’s access to mental health care following incarceration may be particularly important given limited behavioral health services in many rural communities.^3^,^4^ Enhancing mental health services in jails may provide another opportunity to improve mental health symptoms, potentially leading to decreases in substance use and impaired driving.

This study has limitations. First, participants were recruited from three rural Appalachian Kentucky jails and met study eligibility criteria indicating they were high risk, which may limit the generalizability of study findings. Second, only baseline variables were used to predict impaired driving post-incarceration; future studies should also consider post-incarceration variables that may contribute to impaired driving. Third, the study did not collect information on the frequency of impaired driving, or the substances involved. Fourth, a 12-month follow-up period was used; however, future research may consider limiting its focus to impaired driving in the days immediately following incarceration when women may be most at risk.^1^ Finally, data were self-reported, and the accuracy of responses could not be assessed.

Despite these limitations, this study is the first to examine impaired driving among women returning to rural Appalachian communities following incarceration. Almost one-fourth of women reported impaired driving, highlighting not only a public health and safety threat to people on Appalachian roadways but also an important opportunity for targeted prevention and intervention for women, who are already at increased risk post-incarceration. Future research should 1) continue to examine impaired driving as a health risk behavior at community reentry to better identify who is most at risk and 2) explore interventions that may more effectively prevent impaired driving, including interventions that increase access to mental health services.

**SUMMARY BOX**

**What is already known about this topic?**

Substance use following incarceration can lead to a range of negative health consequences, including substance-related infectious diseases and overdose death. Women may be particularly vulnerable during community reentry.

**What is added by this report?**

No existing studies have examined impaired driving as a health risk behavior for individuals returning to Appalachian communities after incarceration. The current study provides preliminary data on impaired driving prevalence among women reentering Appalachian communities after incarceration and identifies substance use, mental health, and criminal legal factors associated with impaired driving.

**What are the implications for future research?**

Study findings suggest that impaired driving should be included as a substance use-related health risk behavior in future community reentry research as well as highlight an important opportunity for targeted prevention and intervention.

## Figures and Tables

**Table 1:** Differences by Impaired Driving Following Incarceration (N=333)

	Impaired Driving		
	Yes (n=76)	No (n=257)	
	Mean (Median) or %	Mean (Median) or %	Effect Size
**Demographic Information**			
Age	31.4 (30.5)	32.7 (32.0)	.164
Race (% White)	98.7	98.8	.006
Education (years completed)	11.3 (11.5)	11.2 (12.0)	−.044
Relationship status (% married/living as married)	34.2	36.6	.021
% employed (6 months prior to incarceration)	23.7	23.0	.007
% had a valid license at baseline	44.7	32.7	.106
**Substance Use Risk (NM-ASSIST)**			
Cannabis	13.6 (10.0)	11.8 (9.0)	−.150
Cocaine^a^	11.7 (6.0)	7.6 (2.0)	−.345
Prescription stimulants	8.0 (2.0)	6.1 (0.0)	−.167
Methamphetamine^b^	19.0 (20.0)	13.4 (5.0)	−.364
Sedatives or sleeping pills^a^	23.7 (28.0)	19.1 (20.0)	−.310
Street opioids	11.8 (0.0)	8.1 (0.0)	−.264
Prescription opioids	30.4 (39.0)	26.9 (33.0)	−.247
**Mental Health (GAIN)**			
% Generalized Anxiety Disorder	48.7	44.4	.036
% Major Depressive Disorder^b^	81.6	64.6	.153
% Post-traumatic Stress Disorder	71.1	64.6	.057
**Criminal Legal System Involvement**			
Number of arrests (lifetime)^a^	3.1 (3.0)	2.7 (2.0)	−.307
Number of incarcerations (lifetime)	5.8 (4.0)	6.1 (3.0)	.028

* NOTE:

a *= p* ≤ .05;

b ≤ .01;;

NM-ASSIST (NIDA-modified Alcohol, Smoking and Substance Involvement Screening Test); GAIN (Global Appraisal of Individual Needs). Effect sizes are reported as Cohen’s D (continuous variables) or Cramer’s V (dichotomous variables).

**Table 2:** Multivariable Logistic Regression Predicting Impaired Driving Following Incarceration (N= 333)

	B	S.E.	*p*	AOR	95% CI
Cocaine NM-ASSIST Score	0.009	0.012	.441	1.009	0.99 – 1.03
Methamphetamine NM-ASSIST Score^a^	0.018	0.009	.047	1.018	1.00 – 1.04
Sedatives/Sleeping Pills NM-ASSIST Score	0.012	0.010	.225	1.012	0.99 – 1.03
Major Depressive Disorder (GAIN)^a^	0.696	0.334	.037	2.006	1.04 – 3.86
Number of Lifetime Arrests	0.147	0.082	.072	1.158	0.99 – 1.36
Impaired Driving in Years Prior to Incarceration	0.125	0.302	.680	1.133	0.63 – 2.05

* NOTE:

a *= p* ≤ .05;

NM-ASSIST (NIDA-modified Alcohol, Smoking and Substance Involvement Screening Test); GAIN (Global Appraisal of Individual Needs).
